# Can nudges save lives?

**DOI:** 10.1007/s42973-021-00095-7

**Published:** 2021-09-07

**Authors:** Fumio Ohtake

**Affiliations:** 1 Center for Infectious Disease Education and Research (CiDER), Osaka University, 2-8, Yamadaoka, Suita, Osaka 565-0871 Japan; 2 Graduate School of Economics, Osaka University, 1-7, Machikaneyama, Toyonaka, Osaka 560-0043 Japan

**Keywords:** Nudge, Heavy rainfall evacuations, Infectious diseases, COVID-19, Behavioral Economics

## Abstract

To assess the promotion of life saving behaviors and determine the sustainability of nudge message effects, this paper examines nudges that promote evacuation during heavy rainfall, preventative COVID-19 infection behaviors, and COVID-19 vaccination. The results showed that altruistic gain messages may have more sustained effects than others in promoting both evacuation during heavy rainfall and contact reduction behaviors as a measure against COVID-19 infection. Specifically, social influence nudges that use a gain frame to convey that a person’s behavior promotes the behavior of others are effective for both heavy rainfall evacuations and encouraging COVID-19 vaccination.

## Introduction

Torrential rain disasters have become an annual occurrence in Japan and other countries worldwide, and many people lose their lives, because they are unable to evacuate before the disaster strikes. To promote evacuation, disaster prevention education has traditionally been used to educate residents on evacuation sites and routes among other aspects. However, even if disaster prevention knowledge increases, many people do not evacuate in the event of an actual disaster. In this context, it is difficult to make evacuation compulsory, because governments cannot fully grasp the location of the residents and it is not realistic to prescribe penalties. Thus, there is a need for policy interventions that can bridge the gap between knowledge and action.

A similar thing is happening in the field of infectious disease control. In 2020, COVID-19 caused many infections worldwide and the behavioral restrictions put in place for infection control had a massive negative impact on the global economy. The requirements involved getting people to avoid going to places with a high risk of infection in addition to basic infection control measures, such as hand washing and wearing masks. In some countries, masks were made compulsory and fines were levied for people who did not wear them, especially when restrictions were in place during severe COVID-19 spreading. Similarly, many countries also imposed restrictions on the operation of restaurants. However, in Japan, even if a state of emergency is declared, based on the Influenza Special Measures Law, businesses can only be requested to close and the general public can only be urged to take infection control measures. Therefore, in this context, it is necessary to have policies that encourage people to take preventive actions against infection in addition to providing knowledge on infection control measures. In other words, if we can create policies that increase the number of people who take desirable actions in situations where direct regulations are not available, such as during disasters or the spread of infectious diseases, we will be able to save people’s lives.

As a potential solution, nudges can bring about behavioral changes in situations where regulations and financial incentives are not available. The application of behavioral economics to public policy is done through these nudges, which Thaler and Sunstein (2008) defined as follows: “A nudge […] is any aspect of the choice architecture that alters people's behavior in a predictable way without forbidding any options or significantly changing their economic incentives” (p. 6). These policy applications of behavioral economics are advancing and are not limited to applications for disaster evacuation behaviors and infectious disease control. Furthermore, Thaler (2018) distinguished between nudge and sludge, the latter being applicable if it makes the person take an undesirable action or an action that is not in their best interest. Ultimately, the purpose is to design a conscientious choice that allows the person to make decisions that they judge to be better.

In particular, Thaler and Sunstein (2008), and Thaler (2018) being awarded the Nobel Prize in Economics in 2017 was a major catalyst. In the UK, behavioral insights teams were established in 2014 for policy applications, while Japan established the Japanese version of the Nudge Unit (BEST) in April 2017. The results of a series of field studies in behavioral economics, such as Madrian and Shea (2001), who found that defaulting on participation in corporate pension plans increased participation, have been incorporated into government policy in the UK private pension system, NEST (Department for Work and Pensions, 2018; Thaler and Benartzi 2004). Scholars have also conducted nudge with messages intervention studies, the most famous and earliest being the Message UK nudge unit field RCT that sent nudge messages in reminder letters to people who had filed returns, but not paid taxes (Hallsworth et al. 2017). In this study, the social norm message that many people pay their taxes on time and those who do not are in the minority was effective. As a result, a variety of nudges are now being adopted as policy tools in countries worldwide, including Japan (OECD 2017).

In another field RCT, conducted in one municipality, Fukuyoshi (2018) examined messages recommending colorectal cancer screening. The one emphasizing that if the patient did not undergo the screening this year, the test kit would not be sent next year increased the screening uptake by 7 percentage points more than a gain-framed message declaring that the test kit would be sent next year if the patient took the screening this year. As another field RCT example that examined one particular municipality, using social comparisons when displaying electricity consumption in the Japanese version of the Nudge Unit (Behavioral Science Team) proved to promote energy-saving behavior (Behavioral Science Team (2018)). Overall, some nudges work and others do not. Some nudges are effective in the short-term, but lose their long-term effectiveness due to habituation. For instance, regarding energy-saving behaviors, Ferraro et al. (2011), and Allcott and Rogers (2014) found that social comparisons have long-term effects, but Ito et al. (2018) discovered that in Japan, nudge messages, reflecting moral calls to save electricity, rapidly lose their effectiveness. However, there is no such effect loss when using financial incentives through dynamic pricing.

Moreover, Sunstein (2015) presented the following eight criticisms of nudges: (1) nudges steer people's choices in certain directions, (2) changing decisions by default is problematic, (3) using anchoring is problematic, (4) people’s preferences are diverse, (5) nudges deprive people of learning opportunities, (6) biases and prejudices exist in governments and bureaucracies that use nudges, (7) nudges distort market competition, as those that favor only certain products have a negative impact on free market competition and reduce people’s incentive to develop new products and services, and (8) warmongering causes people to lose their independence.

However, Sunstein (2015) also addressed these criticisms as follows: (1) nudges are assumed to ensure freedom of choice and people’s actions are called slights when they are aimed toward a particular company’s profit gains (Thaler 2018), (2) many choices already use defaults in the form of opt-ins (e.g., the application-based system in receiving social security benefits and the willingness to donate organs), (3) one particular choice will inevitably come first, no matter the display method, justifying the process of devising a way to make choosing the better option easier; (4) nudges are based on the premise that freedom of choice is guaranteed, but this freedom may not necessarily lead to greater satisfaction for the individual, as in the case of bias and excessive choices; (5) there is anyway little opportunity for learning when making new decisions (e.g., those that are not made many times in life) and some nudges can provide opportunities for learning, (6) imposing transparency and accountability on governments and bureaucracies that use nudges can address their bias and prejudice, (7) nudges should be used to promote market competition when markets fail, due to externalities or monopolies; and (8) people do not make informed and independent decisions on everything, but following rules and habits saves time and energy, and thinking about more important issues allows for independent decision-making.

Thus, based on Sunstein (2015), we can discuss the pros and cons of using nudges to promote evacuation in heavy rainfall disasters and prevent infections. In both cases, at least under Japanese law, it is impossible to enforce measures with penalties^1^. As it is difficult to accurately identify the target population, it is also difficult to use economic incentives, such as penalties for those who do not evacuate or take infection control measures, or subsidies for those who do comply. In addition, it is difficult to make calm judgments in times of disaster or infectious disease epidemics, making it necessary to provide information that takes such factors into account.

In the case of infectious diseases, people may consider the risk of their own infection, but they do not completely consider the risk of infecting others through their actions. Similarly, during a disaster, one’s own evacuation behavior influences the actions of the people around them (Ohtake et al. 2020). The existence of such externalities provides a rational reason for using nudges. Furthermore, evacuation and infection control encompass life-threatening situations that are difficult to solve by simply experiencing them repeatedly and improving them through learning, justifying the use of nudges in related policies. Overall, the question that remains is whether there are nudge messages that can induce behavioral changes.

In this paper, we present our empirical research on the effectiveness of nudge messages in these fields. Specifically, I assess whether nudge messages can promote evacuation behavior during disasters and preventive behavior against infectious diseases. In Sect. 2, I present Ohtake et al.’s (2020) study results on evacuation facilitation nudges during heavy rainfall disasters. Sect. 3 offers a series of studies on effective nudge messages for behavioral changes and encouraging vaccination against novel coronavirus infections (Sasaki et al. 2021; Sasaki, Saito, and Ohtake 2021a, 2021b). Finally, Sect. 4 discusses the conclusions and policy implications.

## Evacuation facilitation nudges during heavy rainfall

### Hypotheses and nudge messages

Ohtake et al. (2020) examined the effectiveness of nudge messages to encourage early evacuation during a torrential rain disaster. Using a questionnaire survey conducted with Hiroshima residents, we analyzed the effects of behavioral economics messages on resident’s evacuation intentions in a hypothetical disaster situation. Based on a follow-up survey, conducted eight months later, we also examined the impact of these messages on long-term disaster prevention awareness and behavior. Hiroshima Prefecture, which suffered a landslide in 2014 that killed 75 people, has been focusing on disaster prevention education and promoting the “Prefectural Citizens’ Collective Movement for Disaster Reduction” as a reflection of the disaster. The goal of this initiative is to enable each prefecture citizen to take appropriate actions to protect their lives from any natural disaster. In addition, it places a particular emphasis on disaster and prevention education. As a result, the percentage of residents who checked into shelters and aimed to take evacuation routes increased significantly from 13.2% in 2015 to 57.2% in 2018^2^. However, only 0.74% of people actually took evacuation actions during heavy rains in 2018. This result suggests the need for policies that go beyond traditional disaster prevention education and determine the psychological aspects of evacuation behavior.

It is possible that people made rational decisions not to evacuate early after receiving correct information about the disaster, but it is also possible that some thought they should evacuate, but failed to take evacuation action. If the latter is applicable, we can increase the number of early evacuees by devising effective ways to provide information. People may weigh the benefits and costs of evacuation (e.g., monetary and non-monetary psychological costs), and only evacuate when the benefits are greater than costs. However, one of the major benefits of evacuation that should be emphasized is avoiding damages from disasters. On the one hand, when deciding to evacuate, people are uncertain that evacuation actions will produce benefits or that damages will occur with evacuation, because whether or not the disaster will actually happen is not certain. On the other hand, evacuation will certainly incur financial and non-financial costs, such as the hassle of traveling to and the inconvenience of living at the evacuation site, and the difficulty of maintaining privacy. As such decisions include making choices through times of uncertainty, behavioral economics biases may inhibit evacuation behaviors.

The following three behavioral economic factors are considered to inhibit evacuation behavior. The first factor is the present bias effect: the time discount rate from the present to the future is smaller than the time discount rate from the future to the further future^3^. This present bias may explain delays in evacuation, as people may decide they will evacuate when an evacuation advisory is issued, but postpone evacuation when the advisory is actually issued, even before heavy rains become severe.

The second factor is the loss aversion effect. When comparing the same gains and losses in absolute value, the magnitude of the change in welfare from the reference point is greater in the case of loss^4^. People’s behavior differs depending on whether they consider evacuation itself to be a troublesome loss or a life-saving gain.

The third factor is diminishing sensitivity: the marginal impact of a gain or loss on the value function declines as the absolute amount of a gain or loss increases. This characteristic leads to asymmetry in the risk aversion of gains and losses. Specifically, uncertain losses are preferred to certain losses (risk-loving), while certain gains are preferred to uncertain gains (risk-averse)^5^. In other words, a person who does not evacuate, despite the possibility of damages, can be interpreted as choosing the risky behavior (uncertain loss: no loss at all or great human suffering), rather than evacuation (certain but small loss).

Accordingly, evacuation behavior is inhibited, due to loss aversion or asymmetry in risk aversion, when evacuation is perceived as a loss. Whether the same factor is perceived as a loss or gain depends on the reference point. In this case, messages that change the point of reference, emphasize the gain of going to a shelter, or highlight the loss of not going to a shelter may change people’s behavior. Furthermore, bottlenecks that inhibit evacuation behavior may involve the way the information is provided and the surrounding people’s behaviors. For example, Kakimoto et al. (2014) showed that calls for evacuation from the police, fire departments, and district officers (public organizations) are effective for people to recognize and act on disaster risks. In other words, the important factors include whether the information is provided directly or not and whether it comes from a trustworthy entity. Similarly, Yasumoto et al. (2018), who studied evacuation behaviors during the 2018 disaster and Typhoon No. 10 in 2017, also showed that many people took evacuation actions, because people around them evacuated (positive externality).

Urata and Hato (2017) developed a theoretical model that incorporates this externality of others’ evacuation behaviors and confirmed its impact by estimating its parameters from data, but this externality can both promote and inhibit evacuation. For instance, if the surrounding people in a certain area do not evacuate, those who sense danger may also not evacuate, lowering the area’s evacuation rate. However, if individuals do evacuate, the people around them will also evacuate, increasing the area’s evacuation rate. This is evidenced in Udagawa et al.’s (2019) questionnaire survey on evacuation intentions during tsunami disasters. They showed that people who believe in the established social norms, such as “people around me think that you should evacuate too in case of a big earthquake”, are more likely to have evacuation intentions. Thus, if social norms and the surrounding people’s behaviors influence other’s evacuation behaviors, then messages that emphasize social norms could also promote these behaviors.

Many studies have used social norms as messages to promote behavioral changes (Hallsworth et al. 2017), tax payments (Larkin et al. 2019), and energy-saving behaviors (Allcott 2011). In the context of evacuations, messages that convey social norms, such as “most of the people around you have already evacuated” or “you are the only one who has not evacuated, yet” are considered to be effective, but they cannot be used in situations where many people do not evacuate. However, messages that show the externalities of one’s own evacuation behavior on that of others, appealing to social norms and altruism, may be effective. Based on the above discussion, the following hypotheses reflect the possible messages to promote early evacuation:

Hypothesis 1 (Social norms and altruism). Providing information that one’s evacuation behavior will promote that of others will encourage evacuation.

Hypothesis 2 (Loss aversion). Messages that emphasize loss will promote evacuation behavior more, compared to those that emphasize gain.

Hypothesis 3 (Diminishing sensitivity). Setting the reference point to the situation of maximum damage and expressing evacuation in terms of gains, rather than losses, will promote evacuation behavior as a safety measure.

Based on these hypotheses, in Ohtake et al. (2020), we created five messages from A to E and added message F, conventionally used in Hiroshima Prefecture, as a control (Table 1). Then, we conducted an RCT on the effect of promoting evacuation through a questionnaire survey.

**Table 1:** Nudge messages used in the intervention

Nudges	Messages
A. Influence gain nudge	In the past, most people who evacuated in response to evacuation orders during heavy rains did so because others around them were evacuating. If you evacuate, you can save the lives of people close to you
B. Influence loss nudge	In the past, most people who evacuated in response to evacuation orders during heavy rains did so because others around them were evacuating. If you do not evacuate, you are putting people’s lives at risk
C. Reference point	When evacuation advisories are issued, due to heavy rains, it is necessary to evacuate as soon as possible. If you must remain at home, just in case, please wear something that can help identify you, as your life may be in danger
D. Gain-framed relief goods	When evacuation advisories are issued, due to heavy rains, evacuating to a shelter will help you secure food and blankets
E. Loss-framed relief goods	If you do not evacuate to an evacuation site when an evacuation order is issued, due to heavy rain, you may not be able to secure food or blankets
F. Control	Every year, a lot of rain falls occur from the beginning of the rainy season, around the start of June, to autumn, due to the influence of rainy season fronts and typhoons. In Hiroshima Prefecture, there have been many disasters, such as landslides, where mountains and steep slopes collapse. We should learn about the damages caused by heavy rainfall, and protect our lives from disasters by developing the ability to make good decisions and take action when danger is imminent

Messages A and B are based on the social norms and altruism of Hypothesis 1. They promote evacuation by increasing people’s awareness of the externality that their own evacuation behavior triggers that of others and by appealing to their altruism. In other words, the message provides information on the social influence of one's actions. By comparing the effects of the two messages (A and B), we can test Hypothesis 2 (loss aversion). Message A promotes evacuation behavior by making the respondent aware of the altruistic benefits of evacuation, and that evacuation will encourage others to evacuate and save their lives. However, message B is framed differently, making the respondent aware that not evacuating will endanger the lives of others and highlighting the loss that may occur by not evacuating.

Although the meanings of the A and B messages are essentially the same, message B is expected to have a stronger influence on evacuation intentions, because of loss aversion^6^. Furthermore, the message in C is based on the behavioral economics property indicating that the degree of risk aversion is asymmetric between the loss and gain phases (Hypothesis 3). It stems from Tierney’s (2005) proposed message, which Thaler (2018) reintroduced: “If you do not evacuate, write your social security number on your body with a magic marker.” The message “Please wear something that can help identify you” has the effect of changing evacuation behavior from the loss phase to the gain phase by changing the reference point. When the reference point is the current situation, that is, the living state, people view evacuation in the loss phase and become risk lovers. In other words, people are more likely to choose a situation where there is a risk of suffering serious damages without evacuating, than to pay the definite cost of evacuation. However, when a person receives message C, they set the reference point to their death and perceive the evacuation behavior in the gain phase. In this phase, people are expected to choose a situation in which they are sure to survive by evacuating, rather than one in which they are at risk of suffering damages without evacuating.

Messages D and E are also expected to be effective in promoting evacuation. For example, Kakimoto et al. (2014) showed that the costs of evacuation travel and spending time in shelters were significant disincentives for evacuation behavior.Therefore, messages that emphasize the gain of going to a shelter or the loss of not doing so are expected to be effective in promoting evacuation, showing people that the cost of evacuation is small. Messages D and E were created for this purpose. Specifically, the message in D emphasizes the benefits of going to a shelter to secure food and blankets, while E conveys the loss of not being able to get supplies without going to the shelter. Although these messages are logically the same, Hypothesis 2 predicts that E, emphasizing loss, will have a greater effect on promoting evacuation.

**Fig. 1:**
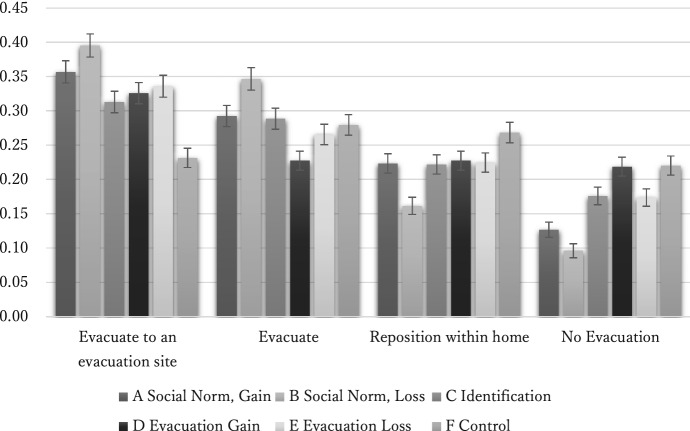
Differences in evacuation intentions by messages

In this context, the purpose of our assessment is to examine whether messages A through E are more effective in increasing evacuation intentions in the short and long term, compared to message F that the Hiroshima Prefecture used to promote evacuation. We also aim to determine which message has the greatest effect and whether the effects of all messages are heterogeneous across targets. Their effects on evacuation intentions are shown in Fig. 1. Based on this graph, messages A and B are effective in increasing evacuation intentions. With the F control message, the percentage of people who answered that they would not evacuate, even if an evacuation order was issued, was 22.0%. However, for people receiving the A message, “Your evacuation will save lives,” the percentage of non-evacuating individuals dropped to 12.7%, and for those who received the B message, “Your failure to evacuate will endanger lives,” this number dropped to 9.62%. Between any two groups, there was a statistically significant difference at the 1% level in the percentage of people who responded that they would not evacuate with messages A, B, and F.

The percentage of those who indicated their intention to evacuate to a shelter also varied greatly among the messages. In the F message, 23.2% responded that they would evacuate to a shelter under the evacuation advisory, but in other messages, the number of people was much higher. The message with the highest number of respondents who would evacuate to a shelter was B (39.5%), followed by A (35.7%). Message E that emphasizes the loss of not going to the evacuation site and message D that highlights the gain of going to the evacuation site was effective for 33.6 and 32.6% of the respondents, respectively. Message C, “wear something that can help identify you,” encouraged 31.3% of the respondents to evacuate to a shelter. In other words, messages A through E, being more statistically significant at the 1% level, are more effective for raising one’s intention to evacuate to a shelter than the control message F.

### Impact on evacuation intentions

Table 2 shows the estimation results using all valid respondents as the sample and one’s intent to evacuate to an evacuation site or not as a non-explanatory variable. Column (1) presents the estimation results of the model with only the messages that are under RCT as explanatory variables, and columns (2) through (4) use the control variables as explanatory variables to account for the possibility that randomization is not complete. Compared to the control F message, the five nudge messages significantly increased the intention to evacuate to a shelter at the 1% level in all estimation models. Among them, message B had the largest effect, increasing the intention by about 15 percentage points compared to the control group. Thus, when people recognized the human characteristic that one will also flee if others around them evacuated, many people understood the externality of their evacuation behavior and responded significantly to the loss that their failure to evacuate would cause to others around them.

**Table 2:** Estimated results on intention to evacuate to shelter

Nudges	(1)	(2)	(3)	(4)
A	0.123***	0.131***	0.116***	0.109***
	(0.0228)	(0.0233)	(0.0285)	(0.0347)
B	0.161***	0.170***	0.149***	0.146***
	(0.0251)	(0.0258)	(0.0310)	(0.0348)
C	0.0816***	0.0832***	0.0681***	0.0610**
	(0.0226)	(0.0215)	(0.0239)	(0.0272)
D	0.0948***	0.107***	0.0788***	0.0799***
	(0.0234)	(0.0226)	(0.0270)	(0.0289)
E	0.103***	0.113***	0.106***	0.0940***
	(0.0251)	(0.0238)	(0.0230)	(0.0280)
Constant	0.233***	0.352***	0.382***	0.206
	(0.0169)	(0.0772)	(0.130)	(0.133)
Observations	5268	4874	2920	2648
*R*^2^	0.011	0.025	0.028	0.044
Number of municipalities	5268	4874	2920	2648
Attribute	N	Y	Y	Y
Household	N	N	Y	Y
Housing	N	N	Y	Y
Trust	N	N	N	Y
Experience	N	N	N	Y
Region	N	N	N	Y
City FE	Y	Y	Y	Y

Robust standard errors are shown in parentheses. ***p < 0.01, **p < 0.05, and *p < 0.1

Next to message B, message A was the most effective, increasing the percentage of people willing to evacuate by about 11 percentage points. Messages D and E were less effective, but they did increase the probability of evacuation by 7.5 percentage points and 10 percentage points, respectively. Based on A, B, D, and E, it appears that people respond more strongly to messages that make them aware of the loss phase. Although omitted from the table, we obtained similar results when analyzing which messages increased the number of people who had the intention to evacuate, regardless of where they evacuated to when an advisory was issued. Specifically, messages A, B, C, and E increased the probability that people would evacuate, while the most effective message was again B.

### Impact of messages on attitudes and behaviors eight months later

#### Follow-up survey

In order to effectively use awareness-raising messages for disaster prevention, these messages must bring about long-term changes in awareness and behavior, and it is necessary to determine which ones have such an effect. As a result, we conducted a follow-up survey in November 2019, using the mail method, with the same 5,598 respondents from the first March 2019 survey. The number of respondents in the follow-up survey was 4,254 (76% collection rate). This follow-up survey asked respondents about their impressions on the multiple messages used in the first survey (A, B, and C), their disaster evacuation and evacuation preparation behaviors, and their evacuation awareness since the first survey.

Specifically, the respondents were asked to respond to “want to evacuate,” “feel a sense of responsibility,” “have room for improvement,” “don’t understand the meaning,” “feel peer pressure,” and “feel repulsion” on a four-point scale (e.g., agree, somewhat agree, neither agree nor disagree, and disagree). Of these, statistically significant differences in the distribution of responses were found between messages A and B for “feeling peer pressure” and “feeling repulsion.” The percentage of respondents who answered “I feel peer pressure” was 8% for message A, 17% for message B, and 12% for message C. The percentage of those who answered “I feel repulsion” was 3% for message A, 11% for message B, and 9% for message C. In all cases, the difference in means between the messages was statistically significant at the 1% level (t-test). In other words, while message B promotes evacuation behavior more than message A, it also makes people more aware of peer pressure and is more likely to make them feel repulsed.

#### Estimation results: descriptive statistics and long-term impact

Table 3 shows the descriptive statistics of the main variables in the follow-up study. A through F indicate which messages were received in the first RCT. The top and bottom rows represent the mean and standard deviation of each variable. When analyzing the long-term impact of a message, we need to consider the fact that Hiroshima Prefecture actually used the A message when the evacuation order was issued. For this reason, we asked the respondents whether they knew of the content in A, “Your evacuation will save everyone’s life,” outside of the first survey [whole message, column (1)]. To analyze the impact on the intention to evacuate and evacuation preparedness behaviors eight months after the message intervention, various questionnaire items were used to test the program’s effectiveness. Statistically significant effects were observed for the five items.

**Table 3:** Descriptive statistics of the follow-up survey

Nudges	(1)	(2)	(3)	(4)	(5)	(6)	(7)
	Hiroshima Prefecture message recognition	Increased evacuation awareness	Evacuation advisory–somewhat or very likely	Always evacuate	Timing	Stockpiling food and water	Emergency preparation
*N*	4210	4220	4221	4221	4188	9722	9667
Whole message	0.406	0.604	0.735	0.239	0.681	0.666	0.720
	(0.0076)	(0.0075)	(0.0068)	(0.0066)	(0.0072)	(0.0073)	(0.0070)
A	0.414	0.627	0.759	0.263	0.706	0.702	0.764
	(0.0188)	(0.0183)	(0.0162)	(0.0167)	(0.0173)	(0.0174)	(0.0161)
B	0.432	0.615	0.734	0.244	0.702	0.677	0.721
	(0.0193)	(0.0189)	(0.0172)	(0.0168)	(0.0179)	(0.0184)	(0.0176)
C	0.406	0.573	0.748	0.230	0.663	0.651	0.715
	(0.0189)	(0.0190)	(0.0166)	(0.0161)	(0.0182)	(0.0184)	(0.0174)
D	0.393	0.620	0.740	0.250	0.663	0.648	0.682
	(0.0182)	(0.0181)	(0.0163)	(0.0161)	(0.0176)	(0.0179)	(0.0175)
E	0.401	0.603	0.721	0.232	0.681	0.676	0.734
	(0.0184)	(0.0184)	(0.0168)	(0.0158)	(0.0176)	(0.0176)	(0.0166)
F	0.392	0.583	0.711	0.219	0.671	0.645	0.705
	(0.0180)	(0.0182)	(0.0167)	(0.0152)	(0.0174)	(0.0176)	(0.0168)

Figures in parentheses are standard errors of the mean

The first variable was “increased evacuation awareness.” The first question was “Has your awareness of evacuation increased since the issuance and announcement of evacuation information using alert levels in June 2019?” Of the responses to this question, 1 was assigned to those who answered “increased” or “increased slightly,” and 0 to those who answered “neither,” “not very much,” or “not at all.” The mean value of this variable was 0.60. Overall, those who received message A had the highest increased awareness of evacuation among each message group.

The second variable was “intention to act when an evacuation order is issued.” This was based on the same question as in the first survey: “If an evacuation order was issued in your area, do you think you would evacuate?” The results are shown in column (3), where “very much” and “somewhat” were counted as 1, and “not much” or “not at all” were counted as 0. The evacuation awareness among those who received message A in the first survey was high (75.0%). The descriptive statistics of the responses to this question, with “very much" as 1 and other responses as 0, are shown in column (4). The third variable was “evacuation timing,” an indicator of commitment to evacuation behavior. Column (5) shows the percentage of respondents who answered that they had decided on some kind of timing. Overall, the percentage of individuals who decided on timing was higher for those who received messages A and B in the first survey.

The fourth variable was “food and water reserves.” This was based on the responses to the question, “Do you currently stockpile at least three days’ worth of food and drinking water?”, where “I stockpile enough” and “I stockpile quite a bit” were set to 1, and “I do not stockpile” or “I stockpile a little” were set to 0. Overall, those who received message A in the first survey tended to stockpile more than those who received control F (Table 3, column 6). The fifth and final variable was “emergency kit preparedness.” A dummy variable was created based on the question “Do you currently prepare emergency supplies other than food and drinking water (portable radio, flashlight, medical supplies, etc.)?” Again, the degree of preparedness of those who received message A in the first survey was higher than that of those who received message F (Table 3, column 7).

To analyze the messages’ long-term impact on consciousness, we employed the same estimated equation as in Sect. 2.2 and conducted it using a municipality fixed effects model. The explanatory variables are female (dummy), age, years of education, income, and number of household members. Table 4 presents the results of this analysis. From columns (1) and (2), we can see that the perception that Hiroshima Prefecture uses message A for publicity does not depend on the type of message distributed in the first survey. This suggests that we can verify the long-term effects of the messages in the first survey from the follow-up survey, because the effects of Hiroshima Prefecture actually using message A are independent of the type of message in the first survey.

**Table 4:** Estimated results on evacuation awareness (follow-up survey)

	(1)	(2)	(3)	(4)	(5)	(6)
	Message recognition		Increased awareness		What to do when an evacuation order is issued	
A	0.0194	0.00479	0.0396*	0.0487**	0.0433**	0.0416*
	(0.0256)	(0.0252)	(0.0223)	(0.0232)	(0.0185)	(0.0205)
B	0.0390	0.0406	0.0308	0.0388	0.0201	0.0280
	(0.0291)	(0.0336)	(0.0270)	(0.0263)	(0.0145)	(0.0168)
C	0.0127	0.00142	0.0121	0.000862	0.0343	0.0361
	(0.0226)	(0.0232)	(0.0223)	(0.0219)	(0.0247)	(0.0270)
D	0.000594	0.00729	0.0336	0.0493**	0.0256	0.0364
	(0.0275)	(0.0277)	(0.0229)	(0.0234)	(0.0205)	(0.0246)
E	0.00786	0.00247	0.0194	0.0234	0.00839	0.0137
	(0.0226)	(0.0252)	(0.0234)	(0.0228)	(0.0193)	(0.0190)
Constant	0.393***	0.162**	0.585***	0.264***	0.713***	0.615***
	(0.0151)	(0.0684)	(0.0138)	(0.0725)	(0.0116)	(0.0619)
Number of observations	4202	3803	4212	3811	4213	3811
*R*^2^	0.001	0.027	0.001	0.013	0.001	0.029
Number of municipalities	30	30	30	30	30	30
Attribute/residence	N	Y	N	Y	N	Y

Robust standard errors are in parentheses

***p < 0.01, **p < 0.05, and *p < 0.1

From columns (3) and (4), message A increased evacuation awareness, compared to message F, by about 3.9 to 4.9 percentage points even when controlling for attributes and residence information (significance level: 5%). In addition, message D increased evacuation awareness more than message F by about 4.9 percentage points when controlling for the same factors (significance level: 5%). Columns (5) and (6) show that message A increased the intention to evacuate by about 4.1 ~ 4.3 percentage points compared to message F, even when the evacuation advisory was issued (significance level: 5 or 10%). These results indicate that messages A and D have a positive effect on long-term evacuation awareness and intention to evacuate.

Finally, we analyzed the message’s long-term impact on specific disaster prevention behaviors. Table 5 presents the estimation results. The results in columns (1) and (2) show that receiving messages A and B increased the probability of deciding when to pre-evacuate by about 3.5 to 3.7 percentage points compared to receiving message F (significance level: 10%). Columns (3) and (4) indicate that receiving messages A and B promoted food and water stockpiling behaviors by about 4 to 5 percentage points (significance level: 10%). Columns (5) and (6) reveal that message A promoted emergency preparedness behaviors by approximately 4.7–5.8 percentage points (significance level 5–10%), but this effect was not observed for message B.

**Table 5:** Estimation results for evacuation preparation behavior (follow-up survey)

	(1)	(2)	(3)	(4)	(5)	(6)
	Determine when to evacuate		Stockpiling food and water		Preparation for emergency items	
A	0.0348*	0.0327	0.0537**	0.0513*	0.0577**	0.0475*
	(0.0197)	(0.0210)	(0.0229)	(0.0256)	(0.0213)	(0.0253)
B	0.0296	0.0374*	0.0322	0.0404*	0.0167	0.0168
	(0.0181)	(0.0211)	(0.0216)	(0.0217)	(0.0187)	(0.0185)
C	0.00723	0.00192	0.00413	0.00848	0.0111	0.00378
	(0.0218)	(0.0215)	(0.0218)	(0.0225)	(0.0273)	(0.0306)
D	0.00986	− 0.000498	0.00106	0.0150	-0.0255	0.0292
	(0.0192)	(0.0170)	(0.0232)	(0.0230)	(0.0250)	(0.0279)
E	0.00861	0.00999	0.0288	0.0359	0.0294	0.0198
	(0.0236)	(0.0230)	(0.0242)	(0.0249)	(0.0193)	(0.0194)
Constant	0.672***	0.458***	0.647***	0.161**	0.705***	0.163***
	(0.0114)	(0.0645)	(0.0144)	(0.0776)	(0.0136)	(0.0580)
Number of observations	4180	3784	4168	3774	4167	3774
*R*^2^	0.001	0.010	0.002	0.021	0.003	0.030
Number of municipalities	30	30	30	30	30	30
Attribute/residence	N	Y	N	Y	N	Y

Robust standard errors are in parentheses

***p < 0.01, **p < 0.05, and *p < 0.1

These results indicate that message A had a significantly positive effect on disaster prevention behavior compared to message F. Therefore, messages that make people aware of gains and disaster prevention are effective in promoting long-term disaster prevention behavior. However, the number of people who actually evacuated in advance, before the disaster that occurred after June, was small. Although not shown in the table, 1882 out of the 4214 respondents (approximately 65%) received an alert level of 3 or higher since June, but only 101 evacuated their homes in advance (approximately 2.4%). There was no difference in pre-evacuation behavior among the messages distributed in the March survey. This may be due to the fact that there were no major disasters, such as the July 2018 torrential rains in Hiroshima Prefecture, during the period from June 2019 to November 2019, when the follow-up survey was conducted, and that there were few evacuation notices issued.

## Effects of nudge messages on countermeasures against COVID-19

### Infection prevention behavior nudges

Many experimental studies have been conducted on nudge messages to promote infection prevention behaviors. For instance, Lunn et al. (2020) found that messages emphasizing the occurrence of an exponential spread of infection were more effective in influencing people to take preventive actions than instructions to maintain a social distance of two meters. Also, Luttrell and Petty (2020) discovered that people find messages that focus on others more persuasive than those that focus on themselves, suggesting that altruistic messages are effective in increasing infection-prevention intentions. Jordan et al. (2020) additionally found that selfish messages, public messages, and a combination of the two are equally effective. However, according to Barari et al. (2020), nudge messages are ineffective for people who are already engaged in preventive behaviors and similar to Everett et al. (2020), that altruistic messages had no effect on behavior. Favero and Pedersen (2020) also reported this ineffectiveness in increasing the intention to take preventive action against infections. On another note, Falco and Zaccagni (2020) revealed that messages emphasizing that infection prevention behaviors are for the benefit of “you and your family” increased intentions to take infection prevention actions, but did not lead to action. Finally, using self-reported behavioral information, Kpran et al. (2021) found that nudge messages may reduce self-reported contact behavior, but they also had the opposite effect on people who originally refrained from contacting others.

In one of my previous publications, Sasaki et al. (2021), we conducted an RCT to find nudge messages to promote infection control measures for new coronaviruses, such as refraining from human contact, masking, and hand washing, using a continuous online questionnaire for the same survey participants. We presented one of the five infection prevention nudge messages in addition to the control message to the same survey participants four times, from April 2020 to August 2020. The four surveys were conducted as follows: Apr 28–30 (1st survey), May 8–13 (2nd survey), Jun 8–12 (3rd survey), and Jul 28-Aug 3 (4th survey). The survey was commissioned to an online survey company called My Voice.com. Overall, we analyzed the answers of the 4241 participants who responded to all four surveys. Immediately after the presentation, we investigated the behavioral intentions regarding infection prevention behaviors and the self-reported infection prevention behaviors in the past week. This study is unique in that it analyzed intentions and behaviors when information was repeatedly provided, rather than one-time behavioral changes. The first two of the four online surveys were conducted while a state of emergency was declared and the other two, after the state of emergency was lifted. Specifically, the first two periods were when the government strongly publicized the need to refrain from going out and traveling during the Golden Week holidays.

We tested the effectiveness of six nudge messages, including a control group:

SKO1 (Control): to prevent infection, reducing contact with others, avoiding the “3 Cs” (closed spaces, crowded spaces, and close contact), practicing proper hand washing, and wearing a mask are effective.

SKO2 (Gain-framed altruistic message): by refraining from going out, avoiding the “3 Cs,” washing your hands, and wearing a mask, you can protect the lives of people close to you.

SKO3 (Loss-framed altruistic message): by going out, not avoiding the “3 Cs,” and not washing your hands or wearing a mask, you will put the lives of people close to you at risk.

SKO4 (Selfish message): by refraining from going out, avoiding the “3 Cs,” washing your hands, and wearing a mask, you can protect your own life.

SKO5 (Altruistic and selfish message): by refraining from going out, avoiding the “3 Cs,” washing your hands, and wearing a mask, you can protect your life and the lives of people close to you.

SKO6 (Simple message): stay home. You can protect the lives of people close to you.

The nudge messages from SKO2 to SKO6 were presented to survey respondents in addition to informational messages about the infection prevention measures used in the control message (SKO1). Prior to presenting the four messages, we measured the extent of avoiding human contact, such as frequency of going to a restaurant or bar, going to work, or traveling, for the week prior to the survey with 10 questions. We also measured the extent of infection prevention behaviors, such as wearing a mask, washing hands, and using a delivery service, with 15 questions. Each question was answered on an eight-point scale, ranging from 0 (never) to 7 (almost every day). We divided the total score for each human contact and infection prevention behaviors by the number of questions (10 and 15) to obtain a behavioral index. After presenting the nudge message, the participants were asked about their intention to take each action. Again, for contact behavior and infection prevention measures, they answered on an eight-point scale, with 0 indicating no intention to stop the behavior and 7 indicating intention to stop definitely. In this case, we also used the mean of the intention related to contact and infection-prevention behaviors as an index.

For contact avoidance behaviors, only the gain-framed altruistic messages had an effect on behavioral suppression in the second survey, but not in the third and fourth surveys. This effect was observed in people who previously had a lot of contact with others. For them, the altruistic gain-framed message had a positive effect on the intention to reduce contact with people every time. Moreover, nudge messages had no effect on promoting infection prevention behaviors. Rather, the gain-framed altruistic message had a negative effect on those who were taking infection prevention behaviors. The loss-framed altruistic message also did not have a statistically significant effect on contact avoidance behaviors, although a larger effect than the gain frame was predicted from loss aversion. However, in the group that did not engage in contact avoidance behaviors, the loss frame did have an effect on raising the intention to engage in these behaviors during the third and fourth surveys. The possible causes for the gain-framed altruistic message’s effect on contact avoidance behaviors in the second survey, but not in the third and fourth are as follows. First, it is possible that presenting the same message repeatedly caused habituation and lost its effect. In particular, the fact that the government, including the expert panel, repeatedly used similar messages may have affected the message. Second, as the second survey was conducted one week after the first survey, it captured the fact that behavioral changes had occurred under the influence of the message. However, there was a month between the second and third surveys, and more than a month between the third and fourth surveys, indicating that the message’s effect only lasted about a week.

In terms of changes in motivation immediately after the message’s presentation, the gain-framed altruistic message had a positive effect on the group that had relatively no contact avoidance behavior until then, both times. Thus, it is possible that a behavioral change occurs for about a week after the message is presented, but that beyond that time, it decreases or disappears. In this case, repeating the message would be effective in maintaining behavioral changes. The loss-framed altruistic message was expected to influence behavioral changes with loss aversion, but as it is difficult to perceive benefits from the message itself, it may not be suitable for repeated behavioral changes and may not have long-term effects in disaster prevention behavior. Nonetheless, this does not mean that loss messages are ineffective for changing behaviors. For instance, Moriwaki et al. (2020) examined messages sent on smartphones and verified individuals’ subsequent behaviors with location information, finding that nudge messages indeed had an effect on behavioral changes. Finally, the most effective message was the one related to economic loss, indicating that the spread of infections would increase unemployment. As in the case of evacuation promotion messages, the loss and gain frames should be based on a comprehensive judgment of whether the effect is a one-time or continuous behavioral change, whether it is large in the short-term or persistent in the long-term, and whether the message is unpleasant.

### Vaccination promotion nudge

#### Survey experiment overview

COVID-19 has a negative externality: it infects the infected person as well as others and worsens their health. In this sense, it is justifiable to use nudges to promote infection prevention behaviors and vaccinations that are effective in preventing the onset of disease and infection. Particularly in the case of vaccinations, due to the risk of adverse reactions, it is necessary to establish a policy that raises vaccination rates, while ensuring individual’s freedoms to not get vaccinated. What kind of nudge message would be effective in increasing the willingness to obtain a vaccine? In this regard, Larkin et al. (2021) found that social norms were associated with people’s trust in the COVID-19 vaccine and Moehring et al. (2021) revealed that communicating the vaccination intentions of others increased people’s willingness to get vaccinated.

In Japan, Tanaka et al. (2021) also conducted a study on nudge messages for COVID-19 vaccinations with 6232 people between the ages of 15 and 59. These individuals were asked about their willingness to obtain the vaccine after reading one of nine nudge messages, including altruistic gain frames, scientific rationales, social norms, and combinations thereof, along with a survey of psychological characteristics. They found that the nudge messages that increased this willingness varied by gender and age group. It was lowest among males in their teens and 20 s, while the social norm nudge motivated males and females in both age groups.

For younger, less prosocial individuals, the motivation to get vaccinated was low, because the selfish benefits of vaccination are very small. Therefore, it is natural that communicating the scientific rationale for vaccination to this group would not be effective. Altruistic messages may increase the motivation to get vaccinated in groups with a high prosocial nature, but cannot be expected to have an effect on those who are not. Even in the case of low prosocial nature, social norm messages may increase motivation to get vaccinated, because they also affect one’s social image, but the effect of nudge messages on younger people remains very small.

Finally, I will discuss two previous studies that I conducted with colleagues, Sasaki, Saito, and Ohtake (2021a, 2021b). We used an online survey experiment method to determine which nudge messages are most effective in increasing vaccination intentions. Although vaccination is the most effective method to prevent COVID-19 infections (Polack et al.(2020); Haas et al.(2021); Fabiani et al. (2021); Thompson et al. (2021); Dagan et al. (2021), in Japan, the start of vaccination was delayed. However, as of May 2021, the vaccination of elderly people aged 65 and above began. Increasing the vaccination rate among the elderly, who are at high risk of serious illness, is very important, because it will alleviate the strain on the healthcare delivery system and lead to a significant decrease in the number of deaths due to infection. Therefore, it is important to clarify how providing information and effective nudge messages affect the vaccination preferences of the elderly.

These studies used two online questionnaires to measure willingness to pay (WTP) for the vaccine when provided with related information. The first questionnaire survey was conducted over a five-day period from Jan 18 to 22, 2021 and the second over a three-day period from Mar 16 to 18, 2021, both among registered monitors of an internet survey company. A hypothetical experimental question was used to measure the WTP for a vaccine to prevent the onset of COVID-19. WTP referred to the maximum amount that one is willing to pay for vaccination. In the first survey, we collected the data by assigning it to match the population distribution of the basic resident register in terms of gender, age, and residence area. In the second survey, we focused on the elderly (65–74-years old), who would be eligible for vaccination at an early stage, and the young sample (25–34-years old), who would be eligible for vaccination later. This data collection process ensured that the number of samples would be evenly distributed in terms of gender and age.

#### Intention to vaccinate by infection status and vaccination progress

We measured the presence or absence of vaccination intention and the amount individuals who were willing to pay or who were willing to receive vaccination in case they were paid for receiving it when the opportunity for vaccination was available, without setting any particular situation. We asked the same respondents about their vaccination intentions in each of the following situations: randomly adding conditions related to “newly infected status” and “vaccination progress.” For the status of new infections, there were two settings: “The number of new infections is decreasing and is at a low level” and “the number of new infections is increasing and is at a high level.” Regarding vaccination progress, there were also two settings: “One out of 10 people of your age living in Japan have already received this vaccine” and “five out of 10 people of your age living in Japan have already received this vaccine.” The respondents were asked to indicate their willingness to be vaccinated in a total of five different situations: no situation setting, two different situations each for infection status and vaccination progress. In addition, the survey was conducted in two settings: (1) the vaccine only has a preventive effect on the onset of infectious diseases and (2) the vaccine also has a preventive effect on infection.

The main results are as follows. The average WTP for the vaccine to prevent the onset of illness among the elderly was ¥2016.5. In this population, 76.4% (seven to eight out of 10) were willing to receive the vaccine if it was offered free of charge. Although the strength of the willingness to get vaccinated varied depending on the situation, most elderly respondents were willing to obtain the vaccine in any situation. Specifically, the intention to obtain the vaccine among the elderly decreased when the number of newly infected people was decreasing and the infection situation calmed, or when the vaccination of people in the same age group had not yet progressed.

The “proportion who will be vaccinated when offered free of charge” and “WTP” factors in the situation where both conditions overlap were the lowest compared to the baseline and other situations where these conditions did not match. Conversely, the willingness to get vaccinated rose in the phase where the number of newly infected people increased or the proportion of vaccinated people in the same age group increased. The latter result supports the possibility that vaccination behavior may have a positive peer effect, encouraging others to get vaccinated. These results were almost the same in both settings, where the vaccine had a preventive effect on disease onset and on infection.

During the first survey, we also examined nudge messages that emphasized selfish and altruistic benefits. Specifically, the nudge message emphasizing selfish benefits was “vaccination protects you from becoming seriously ill” for vaccines with a preventive effect and “vaccination protects you from infection” for vaccines that also prevent infection. To emphasize the altruistic benefits, we used “vaccination helps save lives by freeing up hospital beds” for vaccines with a preventive effect and “vaccination helps control infectious outbreaks and save many lives” for vaccines with infection-preventing effects. However, these messages had no effect on either the intention to get vaccinated or the amount the population was willing to pay.

#### Social comparison and social impact messages

Based on the fact that progress in vaccination status was affected in the first survey, the second survey used the results of the first to examine the effects of social norm messages on vaccination intention and WTP. The three types of messages were as follows:

SSO1 (Comparison nudge): seven to eight out of 10 people in your age group answered that they would receive this vaccine.

SSO2 (Influence-gain nudge): the more people receive this vaccine, the more people have the intention to do so. Your vaccination behavior can encourage the vaccination behaviors of those around you.

SSO3 (Influence-loss nudge): the more people receive this vaccine, the more people have the intention to do so. If you do not receive the vaccine, the people around you may also not do so.

At the baseline, 84.4% of the elderly wanted to be vaccinated and the average WTP was 427.1. The increase in willingness to get vaccinated, compared to the first survey, can be attributed to the fact that the vaccination of healthcare workers had started and vaccine uncertainty had been reduced. However, the average WTP was much lower than in the first survey, potentially because the people whose WTP was originally negative had a desire to get the vaccine and the possibility for adverse reactions was clearly stated in the message. The effects of the nudge messages were as follows. First, “your vaccination will encourage others to be vaccinated” (gain-frame message) strengthened the vaccination intentions of older respondents, who previously had no intention of being vaccinated, and increased the number of those who wished to be vaccinated. Second, “if you do not get vaccinated, others around you will not get vaccinated” (loss message) and “seven to eight out of 10 people in the same age group said they will get vaccinated” (social comparison message) further strengthened the intentions of older respondents, who originally intended to get vaccinated. The results simultaneously suggested that the loss message may place an emotional burden on people and that the social comparison message may weaken the intentions of older respondents who did not intend to be vaccinated. Third, these nudge messages did not have a statistically significant facilitative effect on vaccination intentions among young people.

## Conclusions

This research examines nudges that promote evacuation during heavy rainfall, behaviors for preventing COVID-19 infections, and vaccinations against novel coronaviruses. All nudges promote life-saving behaviors and all three evaluated situations involve externalities in which people's actions affect the actions and health of others. Social norm nudges that emphasize majority behaviors are known to be highly effective, but only when socially desirable behaviors are applied by the majority. When the desirable behavior is in the minority, nudges that turn it into the social norm are also necessary. Overall, this study’s greatest contribution lies in the finding that social influence nudges, in which a gain frame conveys the fact that a person’s behaviors promote those of others, are effective for both evacuation during heavy rains and vaccination against COVID-19.

Furthermore, we examined the persistence of nudge message effects, a common issue in this field. The results suggest that altruistic gain-framed messages may have more sustained effects than others, both in promoting evacuation during heavy rainfall and contact reduction behaviors as a measure against COVID-19. Behavioral economics often emphasize loss messages, because of their loss-aversion properties. However, there is a possibility that the gain frame’s effect is sustained when the message is repeatedly presented, requiring a change in habits rather than a one-time behavioral change.

When using nudges for public policy, multiple nudge messages are possible, based on the theoretical predictions of behavioral economics. However, the average effect and heterogeneity of the target are often unknown until they are actually tested and some messages may be psychologically taxing. Thus, it is important for governments to pretest messages for effectiveness and psychological burden levels before using them.

## Footnotes

1 In February 2021, a partial revision of the Act on Special Measures against Pandemic Influenza established penalties for infected people who refuse to be hospitalized, run away from the hospital, or do not respond to infection control measures, such as active epidemiological surveys.

2 All figures are from Hiroshima Prefecture’s annual survey on prefectural residents' awareness of disaster prevention and mitigation.

3 The present bias is defined as the utility U(c,t) obtained from consumption c at time t, where the time discount rate −[dU(c,t)/dt]/U(c,t) is smaller when t > 0 than when t = 0. Procrastination behavior is also expressed as −[d|U−(−c,t)/dt]|/|U−(−c,t)| _(t=t1)_ > −[d|U−(−c,t)/dt]|/|U−(−c,t)| _(t=t2)_ when t_2_ > t_1 (_> 0).

4 Loss aversion can be mathematically expressed as follows. Specifically, the magnitude of the change in welfare from a reference point is greater in the case of a loss than in the case of the same gain and loss in absolute value. This can be expressed as v′ (for Δx > 0) < v′ (or Δx < 0) for the value function v(Δx) in prospect theory if Δx is the change in welfare from the reference point.

5 The fact that the attitude toward danger differs between gain and loss can be expressed as follows: when Δx < 0 (loss phase), the risk aversion is -v ″/v′ < 0, and when Δx > 0 (gain phase), the risk aversion is -v ″/v′ > 0.

6 Both messages are similar to Katada and Kanai’s (2016) “Be a Leading Evacuee” principle, known as the “Miracle of Kamaishi” as it has been successful in disaster education. Katada and Kanai (2016) explained this principle as follows: *“Be a leading evacuee” does not solely mean to “evacuate first,” but also to “understand the psychological characteristics of people who cannot evacuate and overcome them. (p.850)” Although it is necessary to take actions, while understanding the difficulty of saving one’s own life, if someone begins evacuating first, it will also encourage those who are hesitant to do the same. Thus, evacuating first can save not only one’s own life, but also the lives of others.* By explaining this externality, prioritizing one's own life simultaneously promotes the surrounding people’s evacuation behaviors who follow social norms, it is thought that the evacuation behavior of many people can be promoted.
